# Bisphosphonates in the Management of Patients with Postmenopausal Osteoporosis; Back to the Future

**DOI:** 10.3390/ph18071068

**Published:** 2025-07-20

**Authors:** Socrates E. Papapoulos, Polyzois Makras

**Affiliations:** 1 Center for Bone Quality, Department of Internal Medicine, Section Endocrinology, Leiden University Medical Center, 2333 ZA Leiden, The Netherlands; 2 Department of Medical Research, 251 Hellenic Air Force & VA General Hospital, 11525 Athens, Greece; pmakras@gmail.com; 3 Department of Endocrinology & Diabetes, 251 Hellenic Air Force & VA General Hospital, 11525 Athens, Greece

**Keywords:** bisphosphonates, bone resorption, osteoporosis, treatment sequence

## Abstract

Osteoporosis is a chronic disease associated with significant morbidity and mortality and requires long-term therapy. Efficacious and well-tolerated treatments are available, but their effect is either short-lived or lost following their discontinuation. The exception is bisphosphonates that reduce bone resorption and turnover, can be administered in regimens ranging from once-daily to once-yearly, and have been shown in randomized clinical trials to reduce the incidence of all osteoporotic fractures, but their effect persists following their discontinuation. This is due to their property of being taken-up selectively by the skeleton and being slowly released following treatment arrest. This property allows the discontinuation of bisphosphonate treatment for different periods of time, the so-called drug holiday, which reduces the risk of rare adverse events while maintaining the effect; an action particularly important for patients at very high risk of fractures for whom sequential therapy with different agents is currently advised. Thus, bisphosphonates, apart from being the treatment of choice for certain groups of patients, are also indispensable for the consolidation and maintenance of the gains of all other treatments, providing, in addition, the opportunity of temporary treatment arrest. Most patients with postmenopausal osteoporosis will, therefore, receive bisphosphonate at some stage during therapy of their disease, regardless of their initial fracture risk.

## 1. Introduction

Osteoporosis is a common disease characterized by low bone mass and structural decay of the bone tissue, resulting in increased bone fragility. Fractures, the main clinical consequence of the disease, are frequent; their incidence increases with age, and they are associated with significant morbidity and deterioration of the quality of life and an increase in the risk of new fractures and mortality [1]. The aim, therefore, of any therapy for osteoporosis is the prevention of fractures in patients who have not yet fractured a bone, or ofthe progression of the disease in patients who have already sustained a fragility fracture. This aim can be largely attained with currently available pharmacotherapies complemented with vitamin D and calcium to correct deficiencies/insufficiencies, and measures to reduce the frequency and impact of falls. Osteoporosis is, however, a chronic disease requiring chronic treatment, and while the efficacy and tolerability of approved medications are established, it is important for the planning of optimal long-term management of patients to consider not only the benefits and limitations of these agents but also their pharmacologic properties and mechanisms of action on bone tissue, as well as regulatory restrictions in the duration of their use. Such considerations are particularly relevant for the bisphosphonates that have properties not shared by any other treatment of osteoporosis. In this short review we focus on the rationale and definition of the current position of bisphosphonates in the management of patients with postmenopausal osteoporosis.

## 2. Treatment Rationale

The definition of osteoporosis, as formulated more than 30 years ago, recognized that low bone mass is not the only determinant of bone fragility and that the strength of the skeleton depends also on other properties of the bone tissue, collectively termed bone quality [2]. As with other materials, the structure and material composition of bone together with its mass will determine its ability to resist structural failure, the clinical expression of which is the fracture. Differently, however, from other materials, the skeleton is continuously renewed by the process of bone remodeling, the sequential action of osteoclasts (the bone-resorbing cells) and osteoblasts (the bone-forming cells). Old and damaged bone is removed by osteoclasts and is replaced in the same location by new osteoblast-produced matrix that subsequently mineralizes. Bone remodeling is controlled by osteocytes, differentiated osteoblasts embedded in the mineralized matrix, which orchestrate the function of osteoblasts and osteoclasts in response to endocrine and mechanical signals, and are targets and mediators of therapeutic interventions [3]. On the other hand, bone modeling is a process critical during growth, in which the activities of osteoblasts and osteoclasts are not coupled, as in remodeling, but occur at different sites and promote the deposition of bone at quiescent surfaces [4]. Modelling-based bone formation, therefore, defines the formation of new bone by osteoblasts at sites that have not been previously resorbed by osteoclasts, whereas during remodeling-based bone formation, new bone is formed at bone resorption sites.

In healthy young adults, bone remodeling is balanced so that the volume of bone removed is equal to the volume of bone deposited. However, in osteoporosis, there is an imbalance between bone resorption and bone formation. This imbalance results from a relative or absolute increase in bone resorption and/or decrease in bone formation. These changes combined with the generally higher rates of bone remodeling of patients [5] lead to loss of bone mass, the deterioration of the architecture of cancellous and cortical bone, and a reduction in the degree of mineralization of the bone matrix [6], all of which provide the rationale for the development of treatments that either reduce bone resorption or stimulate bone formation [7].

## 3. Approved Treatments

Most approved agents for the pharmacological management of osteoporosis reduce osteoclastic bone resorption to different degrees and include selective estrogen receptor modulators, bisphosphonates, and the inhibitor of RANKL denosumab. The decrease in bone resorption induced by these agents is invariably followed by a decrease in the rate of bone formation, leading to an overall lower rate of bone remodeling. These changes have beneficial effects on bone strength by reducing the remodeling space, maintaining or, sometimes, improving trabecular and cortical architecture, correcting the hypomineralization of bone tissue, and increasing mineral density [8]. The clinically relevant outcome is the reduction in the risk of fractures. Inhibitors of bone resorption can be provided orally or parenterally in regimens ranging from once-daily to once-yearly administration, ensuring a unique flexibility in the management of patients with osteoporosis compared to those with other chronic diseases. Moreover, long-term (up to 10 years) efficacy and safety information has been obtained in clinical trials with the most potent agents (bisphosphonates and denosumab) [9,10,11,12,13].

Bone-forming therapeutic agents include the agonists of Parathyroid Hormone 1 Receptor (PTH1R), teriparatide and abaloparatide, and the inhibitor of sclerostin romosozumab. Teriparatide and abaloparatide bind to the common G protein-coupled receptor PTH1R in cells of the osteoblast lineage, inhibit their apoptosis, maintain osteoblast numbers, and increase the formation of bone lining cells [14]. In addition, they reduce the production of sclerostin and stimulate the release of RANKL by osteocytes [3,14,15]. They thus stimulate bone formation and also bone resorption, and their activity is mainly due to the stimulation of remodeling-based bone formation. Teriparatide and abaloparatide are administered by daily subcutaneous injections usually for up to 24 months [16,17]. Romosozumab, a humanized antibody against sclerostin, has dual action; it stimulates primarily modeling-based bone formation and inhibits bone resorption [18,19,20]; romosozumab is given by monthly subcutaneous injections for 12 months [21,22,23].

While the magnitude of the bone protective action varies among therapies for osteoporosis, any benefit is invariably and progressively lost at different rates following discontinuation of their administration [18]. The exception is the bisphosphonates, the effect of which is generally maintained for different periods of time following their discontinuation. This is due to their property for specific uptake and longtime retention in the skeleton.

## 4. Bisphosphonate Mechanism of Action

**Figure 1:**
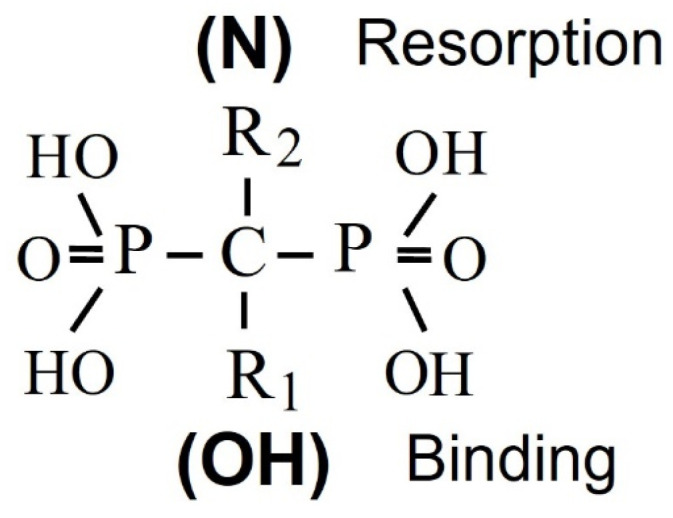
Chemical structure of geminal bisphosphonates. A hydroxyl group in R1 increases the binding affinity of the bisphosphonate to bone mineral, while a nitrogen atom in R2 determines its mechanism of action on bone resorption.

**Figure 2:**
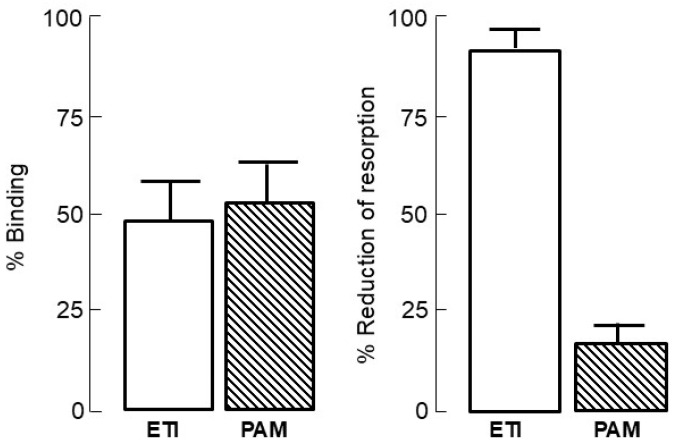
(**Left Panel**) % binding of labeled bisphosphonate on 18d old fetal mouse radii and ulnae in the presence of cold bisphosphonates ETI (Etidronate) and PAM (Pamidronate) 5 × 10^−5^ M; note the similar binding to bone of the two bisphosphonates. (**Right Panel**) % reduction of ^45^Ca release from 17d old fetal mouse metacarpals by ETI and PAM 5 × 10^−6^ M; note the superior antiresorptive potency of pamidronate. Data from ref. [26].

Bisphosphonates, synthetic analogs of inorganic pyrophosphate, are resistant to biological degradation and, hence, suitable for clinical use; they are chemically characterized by their two side chains R1 and R2, respectively, that allow the synthesis of many analogs with different pharmacological properties. A hydroxyl substitution at R1 enhances the affinity of bisphosphonates for calcium crystals, while the presence of a nitrogen atom in R2 enhances their potency and determines their mechanism of action (Figure 1). The whole molecule is responsible for the action of bisphosphonate on bone resorption and probably also for their affinity for bone mineral [24]. The intestinal absorption of bisphosphonates is poor (less than 1%) and decreases further in the presence of food, calcium, or other minerals which bind them. Bisphosphonates are cleared rapidly from the circulation; about 50% of the administered dose concentrates in the skeleton, while the rest is excreted unmetabolized in urine [25]. Their skeletal uptake depends on the rate of bone turnover and renal function, as well as on their structure. Initial studies of the mechanism of action of bisphosphonates on bone resorption focused on their physicochemical properties, but these could not explain their antiresorptive effects, which are rather due to direct effects on osteoclasts [26,27] (Figure 2). 

**Figure 3:**
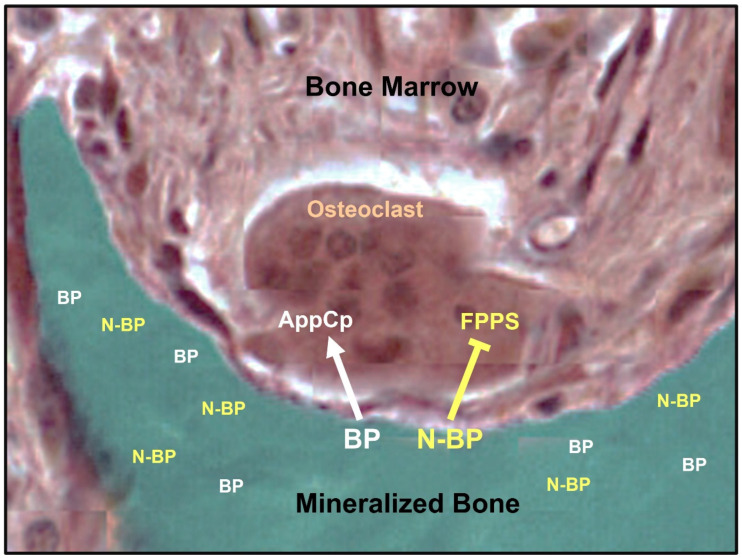
Schematic presentation of the mechanism of action of bisphosphonates on bone resorption. Bone-bound bisphosphonates are released and are taken up by the osteoclast; simple, non-nitrogen bisphosphonates (BPs) are connected to metabolites that induce osteoclast apoptosis, while nitrogen-containing bisphosphonates (N-BPs) inhibit FPPS (farnesyl pyrophosphate synthase), an enzyme of the mevalonate pathway followed by biochemical changes that lead to the inactivation of osteoclasts (see text for details).

Following their administration, bisphosphonates are selectively taken up by bone, preferentially at sites of increased bone remodeling, are released in the acidic environment of the resorption lacunae under the osteoclasts, and are taken up by them [28,29]. After completing their action on osteoclasts on the surface, they are embedded in bone for long periods of time and are slowly released following treatment arrest [24,25]. Non-nitrogen containing bisphosphonates (e.g., etidronate, clodronate) are incorporated in osteoclasts into ATP and are converted to metabolites like AppCp-type nucleotides, which induce osteoclast apoptosis [30,31,32] (Figure 3). Nitrogen-containing bisphosphonates (N-BPs) (e.g., alendronate, ibandronate, pamidronate, zoledronate) inhibit farnesyl pyrophosphate synthase (FPPS) [33] (Figure 3), an enzyme of the mevalonate biosynthetic pathway [34], and induce changes in the cytoskeleton of osteoclasts, such as loss of raffled border, disruption of actin rings, and altered vesicular trafficking, leading to their inactivation and potentially apoptosis [35]. The mechanisms that link the inhibition of FPPS to osteoclast inactivation have been extensively studied and were recently reviewed by Rogers and colleagues [29]. In brief, the inhibition of FPPS by N-BPs prevents the geranylgeranylation of proteins essential for osteoclast function, interfering with the synthesis of lipids required for the post-translational prenylation of proteins that are important for the function and survival of osteoclasts. The simultaneous dysregulation of small GTPases in osteoclasts contributes to the lack of their ability to resorb bone. Importantly, there is a close relation between the degree of inhibition of FPPS and the antiresorptive potencies of N-BPs [36] due probably to differences in the position or orientation of the Nitrogen atom relative to the phosphonate groups required for optimal binding to FPPS [37]. The inhibition of FPPS leads also to the accumulation in blood monocytes of metabolites upstream of FPPS, such as isopentenyl/dimethylallyl diphosphate [38], which activate γδ T cells and stimulate their proliferation. These changes in turn result in an increase in the production of proinflammatory cytokines [39,40] responsible for the acute phase response observed following first exposure, mainly to intravenous administration of N-BPs. In vitro studies reported that inhibition of HMG-CoA reductase, an enzyme higher up in the mevalonate pathway, by statins blocks the synthesis of isopentenyl/dimethylallyl diphosphate and may abolish the activation of γδ T cells [41]. This observation, however, was not confirmed in patients treated with different statins [42,43].

## 5. Bisphosphonate PK/PD Relevant to Long-Term Use

**Figure 4:**
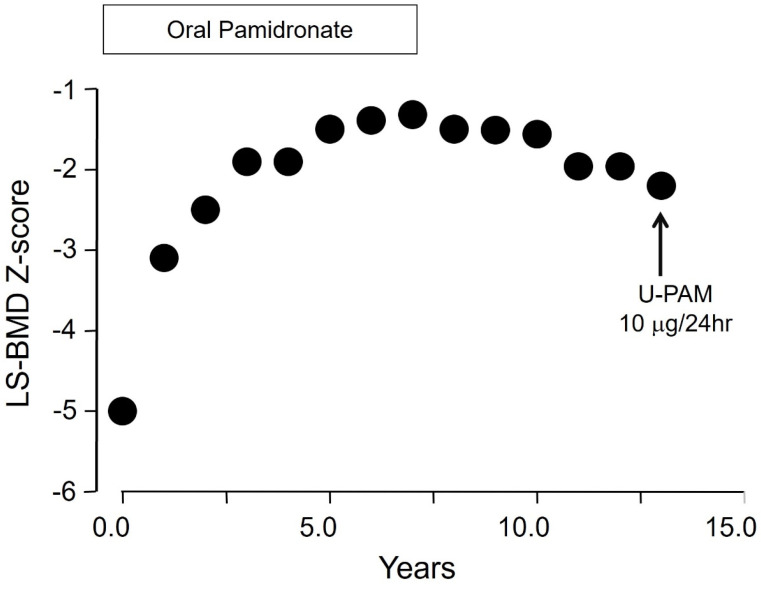
Changes in Lumbar Spine BMD (Z-scores) of an 11-year-old girl with primary osteoporosis during and after treatment with oral pamidronate. Note the very slow decline of the BMD gain in association with the measurable concentrations of pamidronate in urine (U-PAM) 6 years after discontinuation of treatment. Reproduced from ref. [45].

The capacity of the skeleton to retain bisphosphonate is large and the saturation of binding sites in bone with the doses used in the treatment of osteoporosis is unlikely, even if these are given for very long periods [44]. For example, assuming an intestinal absorption of oral bisphosphonates of 0.7% and renal excretion of 50%, N-BPs at doses approved for the treatment of postmenopausal osteoporosis (oral alendronate 10 mg/d, risedronate 5 mg/d, ibandronate 2.5 mg/d, and intravenous zoledronate 5 mg once-yearly) could be administered continuously for at least 47 years (alendronate) and up to 240 years (zoledronate) without any evidence for reduced retention of the bisphosphonate in the skeleton [45]. Thus, saturation of the skeleton with bisphosphonate in clinical practice, an early concern in bisphosphonate development, is impossible. These pharmacokinetic characteristics of bisphosphonates differentiate them from all other agents used in the treatment of osteoporosis and help to interpret their effects on bone tissue, providing at the same time a very powerful tool for the long-term management of patients. For example, in early studies of women and men with osteoporosis and vertebral fractures, cessation of long-term (6.5 years) treatment with daily oral administration of the N-BP pamidronate was not associated with decreases in bone mineral density of the spine and the femoral neck, and the rate of fractures remained stable during 2 years of follow-up without bisphosphonate [46]. Moreover, during this follow-up period, the bone resorption marker urinary hydroxyproline increased but did not return to pretreatment levels due probably to the slow release of embedded bisphosphonate, as shown later by direct measurements of pamidronate in children with osteoporosis in whom the bisphosphonate could be detected in urine for at least 8 years after discontinuation of long-term treatment [47]; an illustrative example of long-term follow-up of a girl with primary osteoporosis treated with oral pamidronate is shown in Figure 4. This general property of bisphosphonates differs among individual compounds used in clinical practice because of differences in their calcium-binding affinity to hydroxyapatite crystals [44]. Thus, early data already indicated that bone surface-bound bisphosphonate is biologically active, whereas the compound that is embedded in the skeleton after completing its action on the osteoclasts is biologically inert and desorbs at very slow rates from the skeleton when treatment is stopped, being responsible for the very slow loss of mineral density after its discontinuation.

## 6. Bisphosphonate and Atypical Femur Fractures

**Figure 5:**
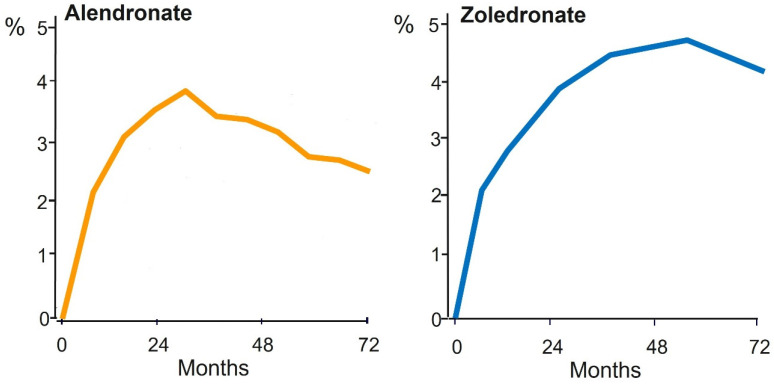
Changes in Total Hip BMD (% of baseline) during treatment of women with postmenopausal osteoporosis with oral Alendronate 10 mg/d (**left panel**) or intravenous Zoledronate 5 mg/yr (**right panel**) in pivotal trials. Note the lack of increases in BMD beyond 3–4 years. Data from ref. [9,60] for Alendronate and Zoledronate, respectively.

Bisphosphonates were, and still are, the most widely used treatments of patients with osteoporosis. Reports, however, of a relationship between unusual low-energy subtrochanteric/diaphyseal femoral fractures (Atypical Femur Fractures, AFF) and long-term treatment with bisphosphonates raised concerns that led to a substantial decline in prescriptions despite their established efficacy and favorable benefit–risk profile [48,49,50,51,52,53]. AFF identified and diagnosed by criteria proposed by a Task Force of the ASBMR [54,55], are rare and are also observed in patients who have never used bisphosphonates. This early observation with radiographic documentation [56] has been repeatedly confirmed and in the most recent analysis of a large cohort in Denmark, 31% of patients with AFF had never received bisphosphonates [57]. Notably, the risk of AFF in bisphosphonate users increases with time, but in patients treated for 3–4 years these are very rare [58]; it was estimated that per 10,000 white women with osteoporosis, after 3 years of bisphosphonate treatment, 149 hip and 541 clinical fractures were prevented, and only 2 bisphosphonate-associated fractures occurred [59]. In addition, the incidence of bisphosphonate-associated AFFs declines rapidly after stopping treatment [58,59]. The risk–benefit ratio is, therefore, highly favorable for BPs given for 3 and up to 5 years. Moreover, in major randomized clinical trials in patients with postmenopausal osteoporosis, bisphosphonates increased hip BMD modestly and this increase reached a plateau after 3 to 4 years (Figure 5), a period when their possible unfavorable effect is minimal, as already discussed. 

Similarly, osteonecrosis of the jaw, currently termed medication-related osteonecrosis of the jaw (MRONJ) as it also occurs with other medications [61,62], is rare in patients with osteoporosis and more frequent in patients with malignant diseases, probably due to the higher doses, frequency of administration of antiresorptive treatments, and co-treatment with other medications predisposing to this adverse event.

## 7. The Drug Holiday

The above-mentioned pharmacologic and clinical properties of bisphosphonates led to the introduction of the concept of drug holiday, the conscious decision to stop using regularly prescribed medications for a period and for a particular reason (Structured Treatment Interruption) to reduce toxicity or tolerance to treatment. Of all treatments for osteoporosis, this concept applies only to bisphosphonates and was included in the advice for long-term treatment by a Task Force of ASBMR [63]. In a recent review, McClung proposed in patients treated for 3–5 years with bisphosphonates to determine whether they still meet criteria for treatment [64]. If they do not, he recommended a bisphosphonate holiday due to the low risk of fracture, followed by BMD measurements at infrequent intervals (2 years after stopping alendronate or zoledronate, 1 year after stopping risedronate or ibandronate, and every 2 years thereafter). If during follow-up the patient meets criteria for therapy once more, bisphosphonate or another agent should be given. If, however, the patient, after the initial course of a bisphosphonate remains at high fracture risk, continuation of treatment to reduce the risk with a different agent should be considered. This approach is based on the above-mentioned considerations but, importantly, also on the evidence of additional increases in BMD by denosumab or a bone-forming agent in patients with postmenopausal osteoporosis previously treated with bisphosphonates [23,65].

## 8. Positioning Bisphosphonates in Long-Term Treatment

The mentioned approach does not imply that the use of bisphosphonates should be restricted to a single period of administration for 3–5 years to selected patients, but it helped to better position bisphosphonates in the management of patients with postmenopausal osteoporosis. While treatments have become available that are more efficacious than bisphosphonates in reducing the risk of fractures, particularly in patients at high risk [22,66], the use of bone-forming agents is restricted to 1 or 2 years for romosozumab and PTH1R agonists, respectively, and their discontinuation is followed by the progressive loss of their effect. Similarly, cessation of treatment with the most potent antiresorptive agent, denosumab, is followed by transient increases in bone remodeling above pre-treatment levels associated with rapid bone loss and increased risk of multiple vertebral fractures [67,68,69,70]. These properties of available treatments have led to proposals to initiate therapy according to the prevalent fracture risk and to apply sequential regimens with different appropriate agents [71]. Sequencing one treatment to another at different ages and stages of disease is an approach that can maximize benefits and avoid potential risks from long-term treatment with a single agent [72]. Bisphosphonates, apart from still being the treatment of choice for certain groups of patients, due to their unique pharmacologic properties, are also indispensable for the consolidation and maintenance of the gains of all these treatments, providing, in addition, important flexibility in the pharmacologic management of postmenopausal osteoporosis that includes the opportunity of temporary treatment arrest.

**Figure 6:**
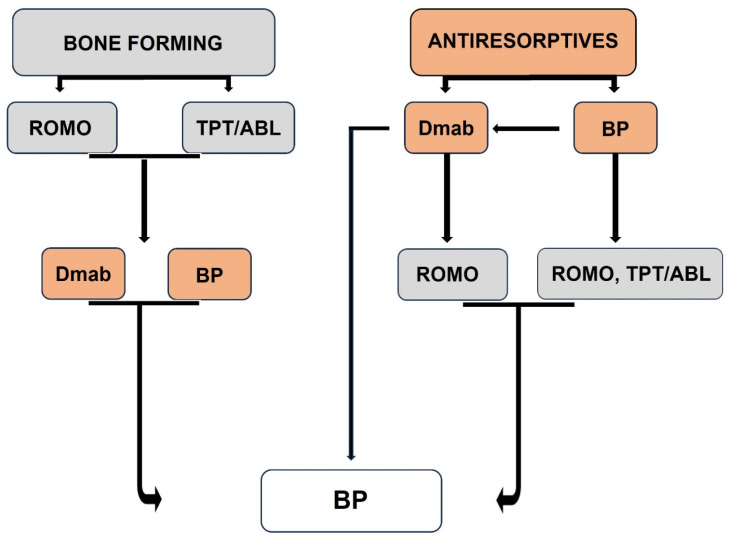
Treatment of women with postmenopausal osteoporosis will include bisphosphonates regardless of their fracture risk. ROMO, romosozumab; TPT, teriparatide; ABL, abaloparatide; Dmab, denosumab; BP, bisphosphonates (Modified from reference [73]).

Thus, based on current evidence, the vast majority of patients with postmenopausal osteoporosis will receive bisphosphonate at some stage during the course of their disease, regardless of their initial fracture risk (Figure 6).

## Data Availability

Not applicable.
